# One‐Dimensional Hydrogen Chains in Li–Hf–H System: A Pathway to High Superconductivity Under High Pressure

**DOI:** 10.1002/advs.202514126

**Published:** 2025-10-31

**Authors:** Kang Yang, Wenwen Cui, Tong Yang, Shicong Ding, Peiheng Lai, Kun Gao, Jingming Shi, Ming Yang, Tong Zhou, Yinwei Li

**Affiliations:** ^1^ Ningbo Institute of Digital Twin Eastern Institute of Technology Ningbo Zhejiang 315200 China; ^2^ Department of Applied Physics The Hong Kong Polytechnic University HungHom Hong Kong SAR 999077 China; ^3^ Laboratory of Quantum Materials Design and Application School of Physics and Electronic Engineering Jiangsu Normal University Xuzhou 221116 China; ^4^ Research Centre for Nanoscience and Nanotechnology The Hong Kong Polytechnic University Kowloon Hong Kong SAR 999077 China

**Keywords:** one‐dimensional hydrogen chain, high pressure, high‐*T*_c_ superconductivity, high through‐put study

## Abstract

The recent discovery of high‐critical‐temperature (high‐*T*_c_) superconductivity in hydrides such as H_3_S and LaH_10_ has significantly advanced the quest for room‐temperature superconductors. This work reports a new class of high‐*T*_c_ hydrides in the Li–Hf–H system characterized by unknown one‐dimensional (1D) hydrogen chains. Through structure prediction methods and first‐principles calculations, the thermodynamically stable compound LiHfH_20_ is discovered, exhibiting a remarkable *T*_c_ of 222 K at 260 GPa. Notably, the distinct 1D hydrogen chains in LiHfH_20_ facilitate strong coupling between high‐frequency phonon modes and hydrogen‐derived electronic states near the Fermi level, significantly enhancing superconductivity. Extending this concept to other *MX*H_20_ compounds (*M* and *X* represent the other 27 elements), it is demonstrated that hole doping further increases the *T*_c_, exemplified by isostructural BeCaH_20_, which achieves a *T*_c_ of 262 K. These findings provide novel and viable high‐pressure hydrides with 1D hydrogen chain for future experimental synthesis toward room‐temperature superconductivity.

## Graphical abstract

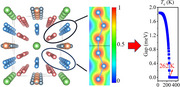

## 1 Introduction

The successful discovery of hydride superconductors through chemical “pre‐compression” has enabled physicists to focus on hydrogen‐rich materials in pursuit of room‐temperature superconductivity.^[^ ^1^ ^]^ Early research on solid H_3_S revealed a remarkable *T*_*c*_ of 200 K,^[^ ^2^, ^3^, ^4^ ^]^ establishing a new class of superconductors‐covalent hydrides. Subsequently, a distinct class of hydrides was identified. These compounds are characterized by three‐dimensional (3D) hydrogen cages that encapsulate metal atoms at their centers. Notable examples include CaH_6_,^[^ ^5^, ^6^, ^7^ ^]^ YH_6_,^[^ ^8^, ^9^, ^10^ ^]^ YH_9_,^[^ ^9^ ^]^ and LaH_10_,^[^ ^11^, ^12^, ^13^, ^14^ ^]^ all of which have been experimentally confirmed to exhibit high‐*T*_c_ values exceeding 200 K under high pressures.

A recent theoretical breakthrough introduced the concept of two‐dimensional (2D) pentagraphene‐like HfH_10_, predicted to exhibit a *T*_*c*_ of 234 K at 250 GPa.^[^ ^15^ ^]^ Analysis of the superconducting properties across these hydrides consistently highlights the critical role of the hydrogen sublattice in achieving high *T*_*c*_ values. These findings continue to inspire theoretical explorations into the properties and configurations of hydrogen sublattices in superconducting systems.

The incorporation of a third elemental degree of freedom into the binary hydrides provides a promising avenue for further investigating the mechanisms underlying the superconducting phase.^[^ ^16^ ^]^ On one hand, the additional element introduces an extra chemical degree of freedom, expanding the structural landscape for identifying candidates with unique hydrogen motifs.^[^ ^17^, ^18^, ^19^, ^20^ ^]^ On the other hand, synergistic interactions between the added element and host compounds can preserve or even enhance the superconducting properties relative to the parent binary compounds. This strategy has been proven highly successful in diverse hydride classes, including covalent hydrides (e.g., CSH_7_,^[^ ^21^, ^22^ ^]^ and LaBH_8_^[^ ^23^, ^24^ ^]^), 3D clathrate hydrides (e.g. Li_2_MgH_16_,^[^ ^25^ ^]^ and LaSc_2_H_24_^[^ ^26^ ^]^), and 2D layered hydrides (e.g. Rb_2_MgH_16_^[^ ^17^ ^]^), enabling either the realization of remarkably high *T*_c_ values near room temperature^[^ ^25^, ^26^, ^27^, ^28^ ^]^ or the stabilization of phases under reduced pressures.^[^ ^19^, ^27^, ^29^, ^30^, ^31^, ^32^ ^]^ Notably, LaBeH_8_ and LaB_2_H_8_ have been successfully synthesized successfully at pressure below megabar pressure, exhibiting *T*_c_ of 110 K at 80 GPa^[^ ^27^ ^]^ and 106 K at 90 GPa,^[^ ^28^ ^]^ respectively.

A compelling question naturally arises: Do one‐dimensional (1D) hydrogen chain configurations exist in hydrides to host exceptional superconductivity, in addition to the established 2D and 3D hydrogen framework? While chain‐like motifs, such as H_7_ chains or analogous structures, have been reported in certain studies,^[^ ^33^, ^34^, ^35^, ^36^ ^]^ achieving a truly infinite 1D hydrogen chain remains significantly challenging. This is primarily due to the highly reactive 1*s* electrons of hydrogen, therefore 1D hydrogen chain demands the presence of additional elements to construct a confined space that prevents hydrogen atoms from aggregating.

As a typical *d*‐block element, hafnium (Hf) possesses an electronic configuration with incompletely filled *d*‐orbitals, which enhances its reactivity in high‐pressure environments. This unique property enables the formation of metastable 1D hydrogen nanotube in HfH_9_^[^ ^34^ ^]^ and nearly stable (≈1–2 meV per atom above the convex hull) 2D pentagraphene‐like hydrogen structures in HfH_10_.^[^ ^15^ ^]^ The possibility of multi‐dimensional hydrogen configuration within Hf‐H system suggests a promising pathway to exploit stronger “pre‐compression” effects and spatial constraints by incorporating a third element, which may stabilize new hydrogen configurations, paving the way for novel high‐pressure hydrogen‐rich materials.

In this study, we identified two thermodynamically stable compounds, LiHfH_20_ and Li_2_HfH_16_, within the Li–Hf–H under high pressures. LiHfH_20_ possesses an ideal 1D infinite hydrogen chain, which can be stabilized above 240 GPa. Electron–phonon coupling (EPC) calculations reveal a high *T*_c_ of 222 K at 260 GPa, driven by strong coupling EPC associated with the 1D hydrogen chains. We further extended our investigation to a broader set of *MX*H_20_ stoichiometries. Among these, BeCaH_20_ emerges as a promising high‐pressure superconductor, exhibiting an enhanced *T*_c_ of 262 K at 260 GPa.

### 1.1 Results and Discussion

**Figure 1:**
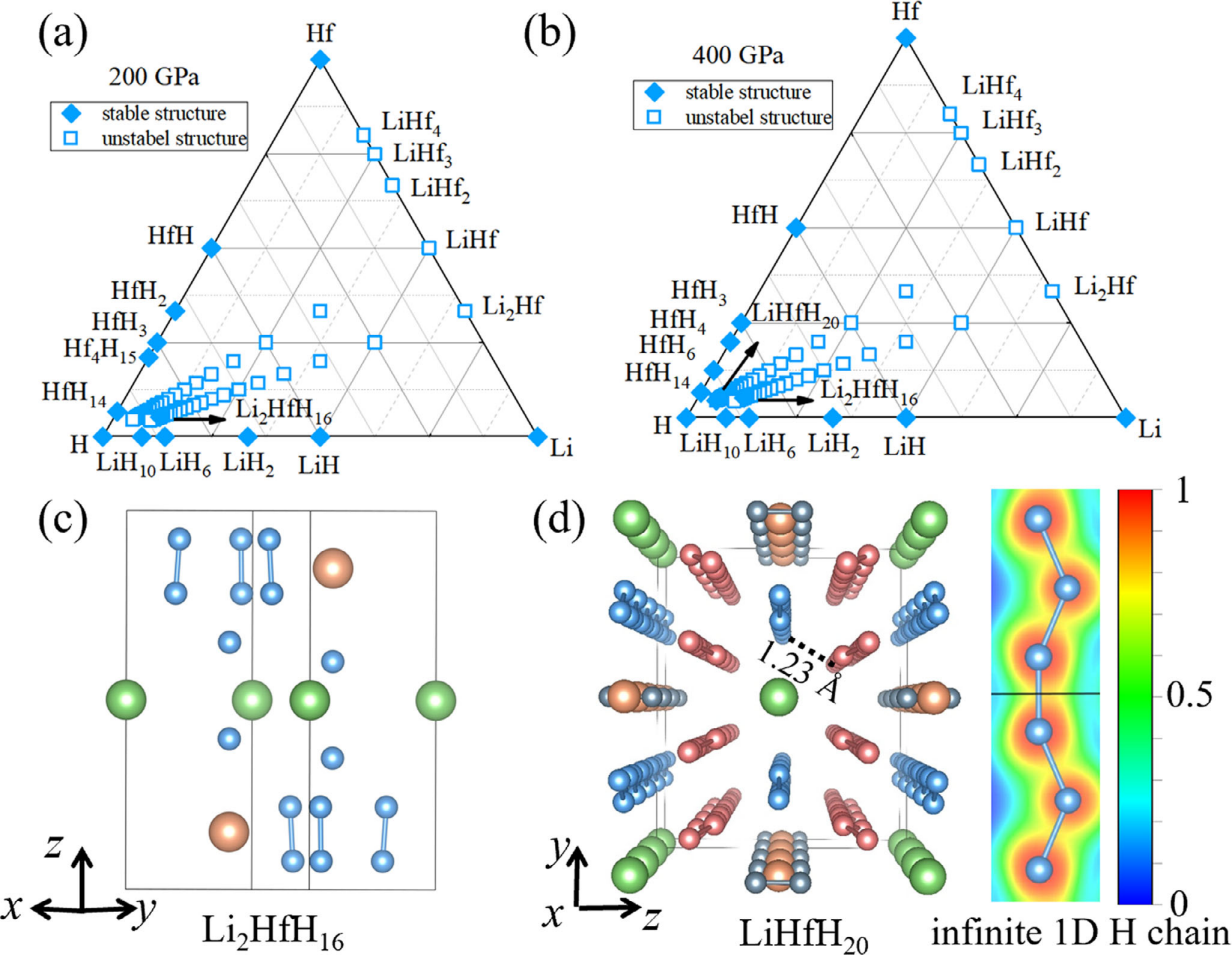
Phase diagram of Li‐Hf‐H system at 200 GPa (a) and 400 GPa (b) Solid rhombus and open squares represent thermodynamically stable and unstable compounds, respectively. Arrows highlight our new predicted compounds. Three‐dimensional view of Li_2_HfH_16_ (c) at 200 GPa and LiHfH_20_ (d) at 260 GPa are shown, including the ELF for the characteristic 1D hydrogen chain. Green and brown spheres represent the metal elements Li and Hf, while other spheres are H atoms. Structural information is shown in Table S1.

We extended our study to the ternary Li_*x*_Hf_*y*_H_*z*_ (*x* = 1–2, *y* = 1–2, *z* = 1–20) systems at pressures of 200, 300 and 400 GPa (see **Figure** **1** and Figure S1, Supporting Information^[^ ^37^ ^]^). Notably, we identified a thermodynamically stable compound Li_2_HfH_16_ at 200 GPa, which remains robust stability without phase transition up to 400 GPa, the highest pressure studied in this work. At 400 GPa, another stoichiometry LiHfH_20_ emerged on the convex hull (see Figure 1b). To determine the stability ranges of these phases, we calculated the relative enthalpy as a function of pressure with respect to possible decompositions (see Figures S2 and S3, Supporting Information). Li_2_HfH_16_ is stable over a broad pressure range from 170 to 400 GPa, while LiHfH_20_ becomes energetically favorable above 390 GPa. The contribution of zero‐point energy (ZPE) is a critical factor in stablilizing hydrogen‐rich compounds due to the high vibrational frequencies associated with the low mass of hydrogen atoms. Incorporating ZPE effects significantly extends the stable pressure range of LiHfH_20_ (Figure S3, Supporting Information), reducing its minimum stable pressure to 240 GPa (Figure S3b, Supporting Information).

The crystal structures of Li_2_HfH_16_ and LiHfH_20_ are shown in Figure 1c,d, respectively, both featuring distinctive hydrogen configurations. The $P\bar{3}m1$ Li_2_HfH_16_ contains isolated hydrogen atoms and H_2_ molecules with covalent H–H bond lengths of 0.86 Å at 260 GPa (Figure 1c). While *Immm* LiHfH_20_ hosts not only conventional H_2_ molecules but also unique 1D infinite hydrogen chains (Figure 1d), which are distinct from the three known structural motifs associated with high *T*_*c*_: 3D cage‐like frameworks, covalent hydrogen networks, and 2D graphene‐like hydrogen sheets. These chains can be classified into two types: type A (pink), which undulate along diagonal planes, and type B (blue), which fluctuate within the (010) plane. The distance between adjacent hydrogen atoms within a chain is ≈1.0 Å, indicating covalent bonding, as confirmed by the electron localization function (ELF) (Figure 1d). This bonding feature supported by a high electron density (1.03 a.u.) and a strongly negative Laplacian of −2.98 a.u. (Table S2, Supporting Information). Conversely, the interchain H⋅⋅⋅H distance is about 1.23 Å, which is too long to support covalent bonding. It is further corroborated by a positive Laplacian value of +2.38 a.u., indicating a closed‐shell interaction. Further Bader charge analysis (Table S3, Supporting Information) reveals that each type A, type B H‐chain and H_2_ molecule in the unit cell receives ≈0.36, 0.38, and 0.08*e* from the metal Hf and Li.

**Figure 2:**
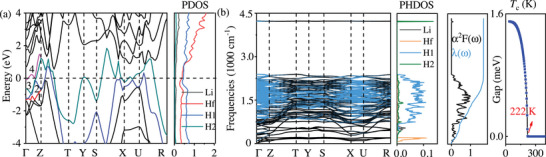
a) Calculated electronic band structure and projected densities of states (PDOS) for LiHfH_20_ at 260 GPa. b) Phonon dispersion, the phonon density of states (PHDOS), eliashberg spectral function, the electron‐phonon strength integral, and isotropic superconducting gap of LiHfH_20_ at 260 GPa. H1 and H2 denote contributions of the 1D hydrogen chain and hydrogen molecules to the DOS (states/eV/cell) and PHDOS, respectively.

To explore the intriguing properties of Li–Hf–H compounds, we compared the electronic and phonon properties of the two identified structures. Both compounds exhibit metallic behavior, with several bands crossing the Fermi energy level (*E*_*f*_) (see Figure S4a, Supporting Information, and **Figure** **2**a). However, the calculated density of states (DOS) reveals distinct contributors to their metallicity. In LiHfH_20_, hydrogen dominates the DOS at *E*_*f*_, contributing 0.49 eV^−1^ per cell and accounting 50% of the total DOS, which is primarily from the hydrogen chains (H1). This is consistent with the significant proportion of hydrogen atoms in the chain configuration. While for Li_2_HfH_16_, the DOS at *E*_*f*_ is mainly attributed by Hf atoms, contributing 0.23 eV^−1^ per cell (Figure S4a, Supporting Information). These findings suggest that LiHfH_20_ is more likely to exhibit a higher *T*_c_ due to the greater H‐derived contribution to the DOS at *E*_*f*_.

As expected, the calculated electron‐phonon coupling (EPC) properties (Figure 2b and Figure S4b, Supporting Information) confirm a significantly higher *T*_c_ for LiHfH_20_ with 222 K at 260 GPa, compared to 95 K for Li_2_HfH_16_ at 200 GPa. While anharmonic effects generally reduce *T*_c_ in compressed hydrides,^[^ ^38^, ^39^ ^]^ the considerable computational cost associated with anharmonic treatments has led us to adopt the harmonic approximation for all calculations reported here. Further analysis reveals that this remarkable difference in *T*_c_ arises from distinct coupling mechanisms. For Li_2_HfH_16_, phonon vibrations in the 200–400 cm^−1^ range contribute 12.8% to the total EPC constant λ, primarily due to pronounced Kohn anomalies and softened phonon modes associated with Li atoms (Figure S4b, Supporting Information). This behavior deviates from the typical characteristics of high‐*T*_c_ superconductors, where hydrogen vibrations are expected to dominate the EPC.

In contrast, LiHfH_20_ exhibits strong EPC contributions from H‐chain vibrational modes in the 500–2600 cm^−1^ range, which account for 74% of the total λ. This results in a significantly larger EPC constant of λ = 1.70 and a logarithmic average phonon frequency ω_log_ = 1217 K, compared to λ = 1.04 and ω_log_ = 887 K in Li_2_HfH_16_. To further analyze the H vibrational modes, we focus on the mode at 1500 cm^−1^ at the Γ point (Figure S5, Supporting Information). This mode involves contributions exclusively from H atoms, and the vibrational direction can either align with the H arrangement in type A or that in type B. We also present the Fermi surfaces corresponding to the colored bands in the Figure 2. Due to the significant degeneracy of these bands, the Fermi surfaces associated with the degenerate bands occupy similar regions within the Brillouin zone (upper panel of Figure S6, Supporting Information), thereby facilitating strong nesting. As a result, pronounced nesting is observed along the high‐symmetry lines Γ‐X and X‐U (bottom of Figure S6, Supporting Information). This finding suggests a strong electron–phonon interaction, further corroborating the high *T*_c_ of LiHfH_20_. The unique 1D hydrogen chain arrangement in LiHfH_20_ enhances the coupling between hydrogen electrons and medium‐frequency phonon modes, playing a crucial role in Cooper pair formation and thereby significantly enhancing superconductivity.

Moreover, we examined the pressure effects on superconductivity through EPC calculations at 320 and 400 GPa. The hydrogen contribution to DOS at the *E*_*f*_ remains similar under these pressures, with values of 0.449 eV^−1^ per cell and 0.459 eV^−1^ per cell under 320 GPa and 400 GPa, respectively (Figure S7, Supporting Information). However the PHDOS reveals notable shifts in hydrogen vibrations with increasing pressure. Specifically, a high‐frequency H–H vibration mode (marked with a red arrow in Figure S8, Supporting Information) shifts upward, indicating an absence of softening modes at higher pressures, which is detrimental to raising *T*_c_. Simultaneously, another range of H‐related vibrations decreases in frequency (highlighted by a blue arrow), reducing the hydrogen contribution to the EPC. As a result, the increased pressure leads to a lower electron–phonon coupling strength, with values of 1.4 at 320 GPa and 1.0 at 400 GPa. While the EPC results indicate an increasing trend ω_log_, rising from 1438 K at 320 GPa to 1636 K at 400 GPa, the *T*_c_  shows a decreasing trend. Specifically, *T*_c_ drops from 222 K at 260 GPa to 209 K at 320 GPa and further to 175 K at 400 GPa (see Figure S9, Supporting Information).

**Figure 3:**
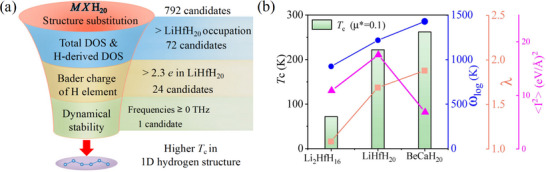
a) The funnel chart illustrates the workflow of the high‐throughput calculations. b) The calculated *T*_*c*_, logarithmic average phonon frequency ω_log_, and λ for Li_2_HfH_16_ at 200 GPa, LiHfH_20_ at 260 GPa, and BeCaH_20_ at 260 GPa.

To further strengthen the electron‐phonon coupling interaction and enhance superconductivity, we trace back to the Hopfield expression and investigate modifications of the *Immm*‐LiHfH_20_ structure. This was achieved by substituting 27 different elements as *M* or *X* (Li or Hf, respectively) to form *MX*H_20_ compounds at 260 GPa (**Figure** **3**a and Figure S10, Supporting Information). Our initial screening focused on the total DOS and the hydrogen‐derived DOS at *E*_*f*_. A total of 72 structures were identified with higher values than the benchmark set by LiHfH_20_, indicating potential candidates for high‐*T*_c_ BCS superconductors. Additionally, we employed the Bader charge of hydrogen as a novel screening criterion, inspired by recent work.^[^ ^25^ ^]^ Among the candidates, 24 structures were identified where hydrogen atoms possess a greater electron density. Finally, we evaluated the dynamical stability of these candidates, identifying one compound, BeCaH_20_, as dynamically stable. In BeCaH_20_, the bond length of the H chains range from 0.88 Å to 1.08 Å. Additionally, a significant interaction between the chains is evident, characterized by a reduced distance of 1.06 Å, illustrated in Figure S11 (Supporting Information). Given its favorable characteristics and adherence to the established criteria, we believe that BeCaH_20_ represents a promising candidate for achieving higher *T*_c_ within the 1D hydrogen‐chain framework.

**Figure 4:**
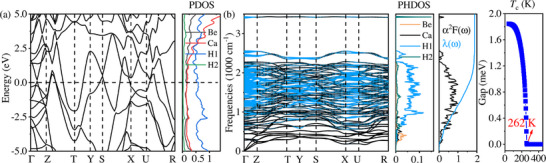
a) Calculated electronic band structure and PDOS for BeCaH_20_ at 260 GPa. b) Phonon dispersion, PHDOS, Eliashberg spectral function, the electron–phonon strength integral, and isotropic superconducting gap for BeCaH_20_ at 260 GPa.

As expected, BeCaH_20_ exhibits an enhanced *T*_*c*_ of 262 K, approaching room temperature with an increased λ and ω_log_ (Figure 3b). The electronic band structure and DOS (**Figure** **4**a) reveal a higher value of 0.62 eV^−1^ per cell and an increased contribution from hydrogen, accounting for 60% of the total DOS. This enhancement arises from a reduction in the average oxidation state of dopants within the hydrogen framework, decreasing from average +2.5 (Li^+^ and Hf^4 +^) to +1.5 (Be^+^ and Ca^2 +^). This shift lowers *E*_*f*_ by approximately 1 eV, resembling hole‐doping, thereby increasing the hydrogen‐dominated DOS, which facilitates Cooper pair formation. Furthermore, the BeCa combination, despite it has lower average oxidation state, leads to an increase in electron transfer to hydrogen, as confirmed by Bader charge analysis in Table S3 (Supporting Information). The charge on hydrogen increases from 2.3 *e* per cell to 2.49 *e* per cell, increasing the H occupation at E_*f*_, which is favorable for raising *T*_*c*_. When we further consider the effect of spin‐orbital coupling (SOC) on the band structure of BeCaH_20_ (Figure S12, Supporting Information), a gap opens at the the high‐symmetry point S. Further simulation of surface states shows no Dirac points here. In BeCaH_20_, the DOS at *E*_*f*_ are mainly dominated by the H atoms, in contrast to LiHfH_20_, where both H and Hf atoms contribute substantially. It is understandable considering the large *d*‐electrons occupation of Hf atoms (see Figure 4a and Figure S13, Supporting Information). Nevertheless, both compounds retain pronounced H‐derived states at *E*_*f*_, which is beneficial for superconductivity. Additionally, phonon frequencies in the range of 500–2500 cm^−1^ provide a dominant contribution to the EPC, accounting for 87.6% of the total coupling (Figure 4b). The EPC constant, λ, increases to 1.88 (Figure 3b), driven by a pronounced peak at 500 cm^−1^. This peak is attributed to softened phonon modes of hydrogen atoms. The combined increase in λ, hydrogen‐projected DOS, and Bader charge on hydrogen synergistically enhances *T*_*c*_ to 262 K. The EPC λ could be also estimated as λ = *N*(*E*_F_)〈*g* ^2^〉/*M*〈ω^2^〉,^[^ ^40^ ^]^ where *N*(*E*_F_), 〈*g* ^2^〉, 〈ω^2^〉, and *M* are the electronic density of states at the Fermi level, the average over the Fermi surface of electron‐phonon coupling matrix elements squared, the average of the phonon frequencies squared, and the atomic mass, respectively. The <*g* ^2^ > of hole‐doped BeCaH_2_0 (6.48 eV^2^) is much smaller than that of LiHfH_20_ (14.68 eV^2^), as shown in Figure 3, indicating that the enhancement of *T*_c_ arises mainly from the increased *N*(*E*_F_). We further investigate the thermodynamic stability of BeCaH_20_ by evaluating its formation enthalpy as a function of pressure (Figures S14 and S15, Supporting Information). Although *P*1 transformed to *Immm* at 350 GPa, BeCaH_20_is energetically unstable under high pressure. While the metastability of these materials presents significant challenges for experimental synthesis, it does not preclude their successful production.^[^ ^41^, ^42^ ^]^ Here, we propose two possible synthetic routes via the chemical reactions: CaH_4_ + BeH_2_ + 7H_2_ and CaH_2_ + BeH_2_ + 8H_2_. In both cases, the calculated formation enthalpies of BeCaH_20_ phase are the negative value across the entire pressure range considered, implying the potential existence.

## 2 Conclusion 

In summary, we have systematically explored the Li–Hf–H system through crystal structure prediction and first‐principles calculations, identifying two thermodynamically stable ternary compounds: Li_2_HfH_16_ and LiHfH_20_, each characterized by distinct hydrogen configurations. Particularly notable is LiHfH_20_, featuring infinite 1D hydrogen chains and exhibiting superconductivity with a high *T*_c_ of 222 K at 260 GPa. This exceptional superconductivity results from strong electron–phonon coupling of 1D hydrogen chain involving hydrogen‐derived electronic states and their high‐frequency vibrations. The 1D infinite hydrogen chain compensates for the diversity of the hydrogen sublattice dimension in hydride superconductors with *T*_c_ higher than 200 K. To generalize this structural model, we conducted high‐throughput calculations on various *MX*H_20_ compounds, finding that BeCaH_20_, a hole‐doped counterpart of LiHfH_20_, further elevates *T*_c_ to 262 K. Our results highlight the significance of innovative hydrogen structures, particularly 1D hydrogen chains, as crucial motifs in achieving high‐temperature superconductivity under high‐pressure conditions, offering valuable insights for future experimental realization of room‐temperature superconductors.

## Conflict of Interest

The authors declare no conflict of interest.

## Supporting information

Supporting Information

## Data Availability

Data sharing is not applicable to this article as no new data were created or analyzed in this study.
