# Supplementary material for: One‐Dimensional Hydrogen Chains in Li–Hf–H System: A Pathway to High Superconductivity Under High Pressure
Source: Adv Sci (Weinh). 2025 Oct 31;12(46):e14126. doi: 10.1002/advs.202514126 (PMC12697900; doi:10.1002/advs.202514126)
Supplement: Supplementary file 1 — Supporting Information [file ADVS-12-e14126-s001.pdf]

Supplementary Materials for "One-Dimensional Hydrogen Chains in Li-Hf-H System: A Pathway to High Superconductivity under High Pressures"

Kang Yang,[1,2,3,*] Wenwen Cui,[3,†] Tong Yang,[2] Shicong Ding,[3] Peiheng Lai,[2]
Kun Gao,[3] Jingming Shi,[3] Ming Yang,[2,4,‡] Tong Zhou,[1,§] and Yinwei Li[3,¶]

[1]*Eastern Institute for Advanced Study, Eastern Institute of Technology, Ningbo, Zhejiang 315200, China*
[2]*Department of Applied Physics, The Hong Kong Polytechnic University, HungHom, Hong Kong SAR, China*
[3]*Laboratory of Quantum Materials Design and Application, School of Physics and Electronic Engineering, Jiangsu Normal University, Xuzhou 221116, China*
[4]*Research Centre for Nanoscience and Nanotechnology, The Hong Kong Polytechnic University, Kowloon, Hong Kong SAR 999077, China*

(Dated: October 24, 2025)

METHOD

To search crystalline structures of Li-Hf-H binary system at 200 GPa, 300 GPa, and 400 GPa, we perform structural prediction up to four formula units by using particle-swarm optimization algorithm CALYPSO [1–4]. CALYPSO is one of the most efficient methods for structural prediction and has been successfully used to predict stable or metastable ground-state structures for various systems at high pressure [5–9]. The following structure relaxations and electronic properties are carried out by using density functional theory implemented in VASP code [10]. The exchange-correlation potential is adopted the generalized gradient approximation (GGA) in the form of the Perdew-Burke-Ernzerhof (PBE) [11] functional. The ion-electron projector augmented wave method is used with valence electrons of $1s^22s^1$, $5p^66s^25d^2$, $2s^2$, $3s^23p^64s^2$ and $1s^1$ for Li, Hf, Be, Ca and H atoms, respectively. The Monkhorst-Pack k-points [12] with a grid density of 0.2 Å^{-1} were chosen to make a total energy convergence of better than 1 meV per atom. To validate the accuracy of PAW pseudopotential under high pressure, we compared the Birch-Murnaghan equation of state obtained with PAW potentials with the full-potential linearized augmented plane-wave method (LAPW) as implemented in WIEN2k [13] (shown in Fig. S16). The cutoff energy for the expansion of the wave function in the plane wave basis was set to 1000 eV. Phonon calculations are carried out using a supercell approach, as implemented in the PHONOPY code. The electron-phonon coupling (EPC) calculations for all structures were performed by using the linear response theory through QUANTUM-ESPRESSO code [14] with ultrasoft pseudopotentials and a kinetic cutoff energy of 100 Ry. The Methfessel-Paxton broadening was set to 0.04 Ry. A q mesh of $6 \times 3 \times 3$ and a k mesh of $24 \times 12 \times 12$ for $LiHfH_{20}$ and $BeCaH_{20}$ in the first Brillouin zone is used in the EPC calculations. A q mesh of $7 \times 7 \times 4$ and a k mesh of $28 \times 28 \times 16$ meshes are used for Li_2HfH_{16}. The convergence the Monkhorst-Pack k-points on the EPC parameter λ was tested using a set of Gaussian broadenings with different k grids, as shown in Fig. S17. The high-throughput screening framework is developed by Dr.Tong Yang.

SUPPLEMENTARY TABLE AND FIGURES

[1] Y. Wang, J. Lv, L. Zhu, and Y. Ma, Phys. Rev. B **82**, 094116 (2010).
[2] Y. Wang, J. Lv, L. Zhu, and Y. Ma, Comput. Phys. Commun. **183**, 2063 (2012).
[3] B. Gao, P. Gao, S. Lu, J. Lv, Y. Wang, and Y. Ma, Sci. Bull. **64**, 301 (2019).
[4] X. Shao, J. Lv, P. Liu, S. Shao, P. Gao, H. Liu, Y. Wang, and Y. Ma, J. Chem. Phys. **156** (2022).
[5] W. Cui and Y. Li, Chin. Phys. B **28**, 107104 (2019).
[6] K. Yang, W. Cui, J. Hao, J. Shi, and Y. Li, Phys. Rev. B **107**, 024501 (2023).
[7] K. Yang, K. Yang, T. Yang, J. He, M. Yang, and T. Zhou, Phys. Rev. B **110**, 054108 (2024).
[8] Flores-Livas, José A and Boeri, Lilia and Sanna, Antonio and Profeta, Gianni and Arita, Ryotaro and Eremets, Mikhail, Phys. Rep. **856**, 1 (2020).
[9] E. Zurek and T. Bi, J. chem. phys. **150**, 050901 (2019).
[10] G. Kresse and J. Furthmüller, Phys. Rev. B **54**, 11169 (1996).
[11] J. P. Perdew, K. Burke, and M. Ernzerhof, Phys. Rev. Lett. **77**, 3865 (1996).
[12] W. Tang, E. Sanville, and G. Henkelman, J. Phys. Condens. Matter **21**, 084204 (2009).
[13] P. Blaha, K. Schwarz, P. Sorantin, and S. Trickey, Comput. Phys. Commun. **59**, 399 (1990).
[14] P. Giannozzi, S. Baroni, N. Bonini, M. Calandra, R. Car, C. Cavazzoni, D. Ceresoli, G. L. Chiarotti, M. Cococcioni, I. Dabo, *et al.*, J. Phys.: Condens. Matter **21**, 395502 (2009).

TABLE S1. Structural parameters of various structures studied in this work.

Pressure (GPa)	System	Space group	Lattice Parameters (Å)	Atom	Atomic coordinates (fractional) x	y	z
200	Li_2HfH_{16}	$P\bar{3}m1$	$a = 2.958$	Li ($2d$)	0.66667	0.33333	0.15169
			$b = 2.958$	Hf ($1b$)	0.00000	0.00000	0.50000
			$c = 5.981$	H ($6i$)	0.82949	0.65899	0.92459
				H ($2d$)	0.33333	0.66667	0.28755
				H (2d)	0.33333	0.66667	0.34743
				H ($6i$)	0.84108	0.15892	0.78157
260	$LiHfH_{20}$	$Immm$	$a = 2.834$	Li ($2c$)	0.50000	0.50000	0.00000
			$b = 5.076$	Hf ($2b$)	0.50000	0.00000	0.00000
			$c = 6.106$	H ($16o$)	0.31893	0.71221	0.85214
				H (4i)	0.50000	0.50000	0.28755
				H ($8i$)	0.00000	0.78732	0.88292
				H ($8m$)	0.17635	0.50000	0.78005
				H ($4g$)	0.00000	0.42717	0.00000
260	$BeCH_{20}$	$Immm$	$a = 2.735$	Be ($2d$)	0.00000	0.50000	0.00000
			$b = 6.276$	Ca ($2b$)	0.50000	0.00000	0.00000
			$c = 5.001$	H ($16o$)	0.31789	0.14197	0.70217
				H ($4i$)	0.00000	0.00000	0.41987
				H ($8l$)	0.00000	0.13072	0.80213
				H ($8n$)	0.66030	0.72347	0.00000
				H ($4g$)	0.00000	0.18822	0.00000

TABLE S2. Topological analysis of covalent bonding about H-H for $LiHfH_{20}$ and $BeCaH_{20}$.

Phase	H-H bond length	$\nabla^2\rho(\boldsymbol{r})$ (a.u.)	$\rho(\boldsymbol{r})$ (a.u.)
$LiHfH_{20}$	1	-2.98	1.03
260 GPa	1.23	2.38	0.65
$BeCaH_{20}$	0.88	-9.2	1.36
260 GPa	1	-2.98	1.03
	1.05	-1.36	0.91
	1.08	-0.06	0.82

* kyang@eitech.edu.cn for managing the manuscripts
† wenwencui@jsnu.edu.cn
‡ kevin.m.yang@polyu.edu.hk
§ tongzhou@eias.ac.cn
¶ yinwei_li@jsnu.edu.cn

TABLE S3. Calculated Bader charge and electronic properties for Li_2HfH_{16}, $LiHfH_{20}$ and $BeCaH_{20}$. Note that there are four chain A (3 H atoms), two chain B (3 H atom) and one molecular H_2 (2 H atoms) in one primitive cell of $LiHfH_{20}$ and $BeCaH_{20}$.

Phase	Bader charge (e)	H-derived DOS	Total DOS
Li_2HfH_{16} 200 GPa	Li +0.83<br>Hf +1.73<br>H -0.21	0.08	0.51
$LiHfH_{20}$ 260 GPa	Li +0.78<br>Hf +1.52<br>H chain A -0.38<br>H chain B -0.35<br>molecular H_2 -0.08	0.49	1.79
$BeCaH_{20}$ 260 GPa	Be +1.6<br>Ca +0.89<br>H chain A -0.42<br>H chain B -0.26<br>molecular H_2 -0.29	0.62	2.05

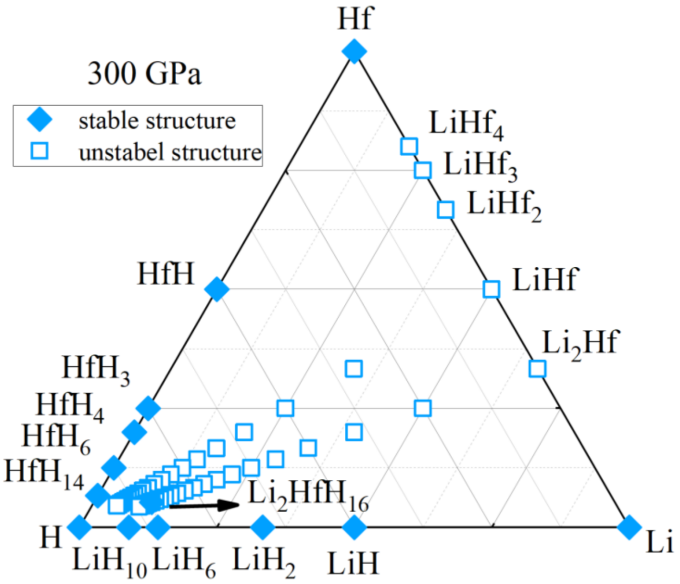

FIG. S1. Convex hull of Li-Hf-H system at 300 GPa. The blue solid symbol represents the thermaldynamically stable phase and the open symbol shows unstable phase.

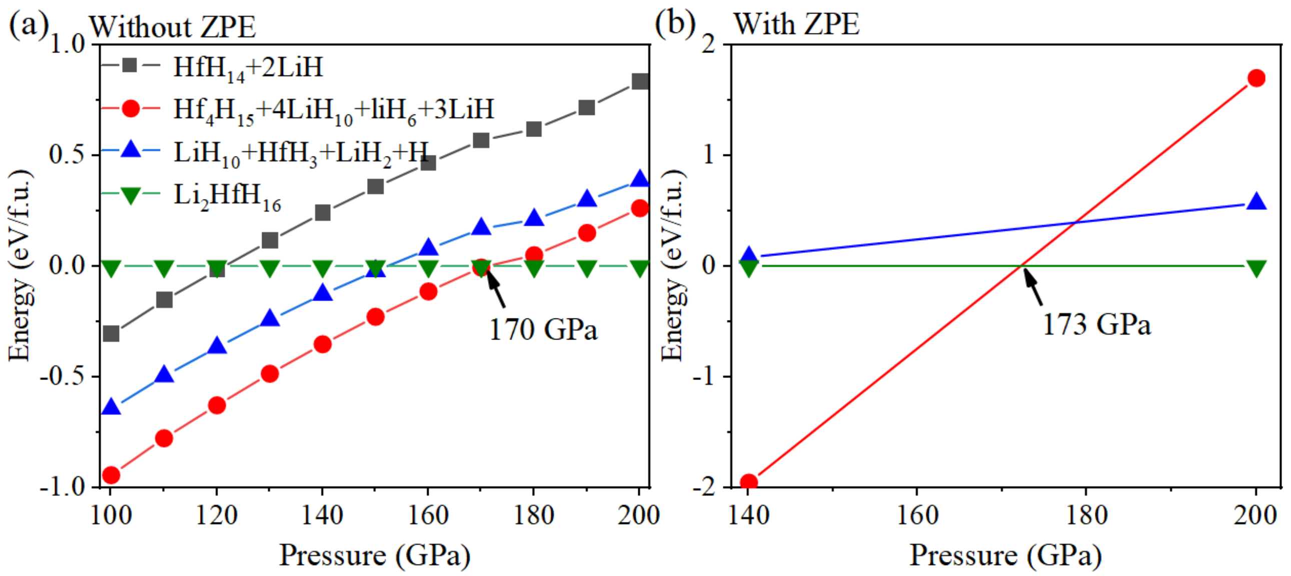

FIG. S2. Pressure dependent variation of enthalpy for Li_2HfH_{16}: (a) Without considering ZPE and (b) considering ZPE.

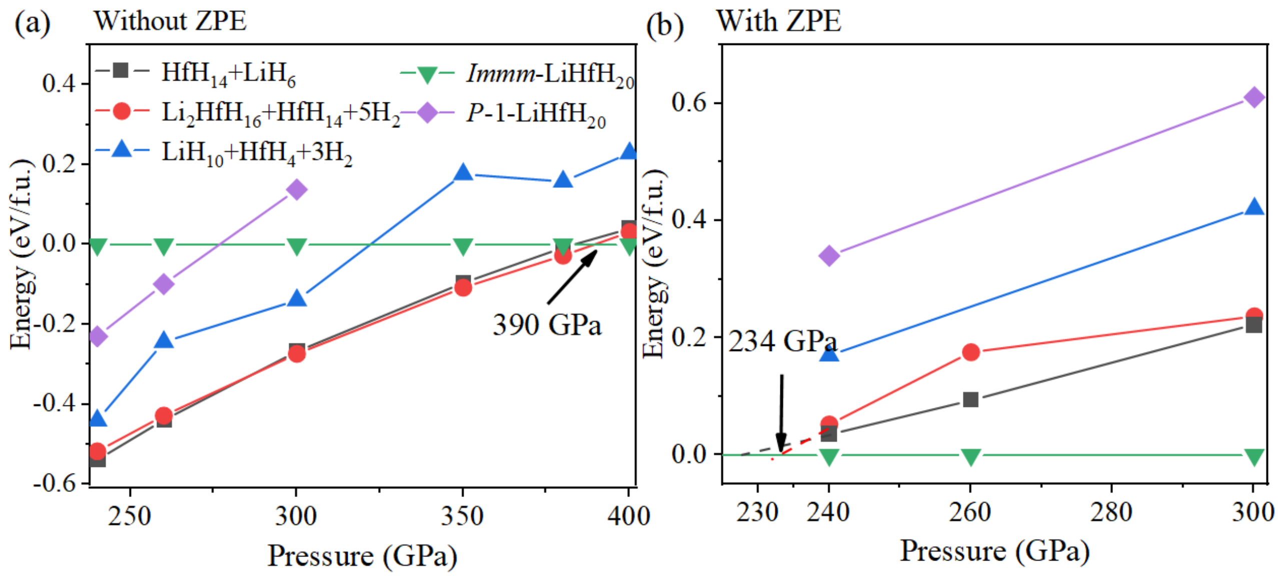

FIG. S3. Pressure dependent variation of enthalpy for $LiHfH_{20}$: (a) Without considering ZPE and (b) considering ZPE.

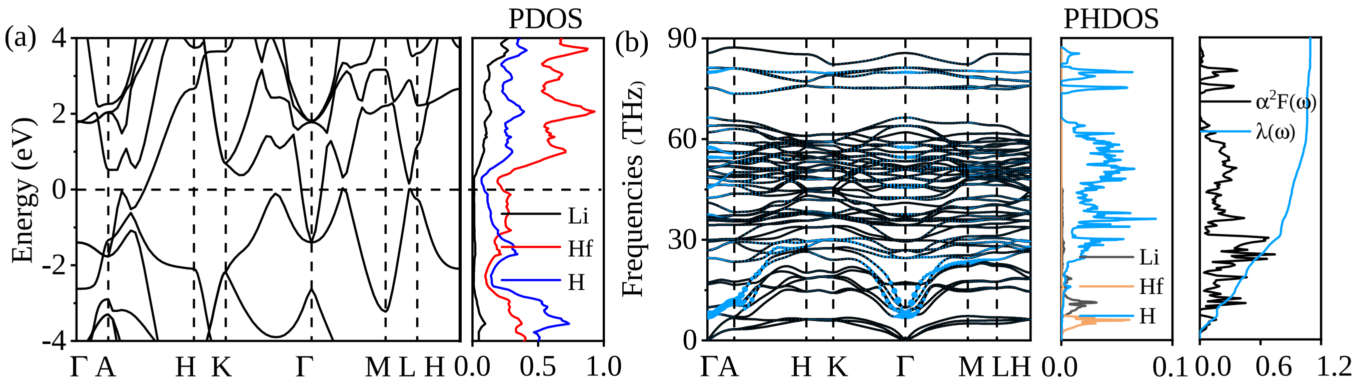

FIG. S4. (a) Calculated electronic band structure and projected densities of states (PDOS) for Li_2HfH_{16} at 200 GPa. (b) Phonon dispersion, the phonon density of states (PHDOS), eliashberg spectral function, and the electron-phonon strength integral of Li_2HfH_{16} at 200 GPa.

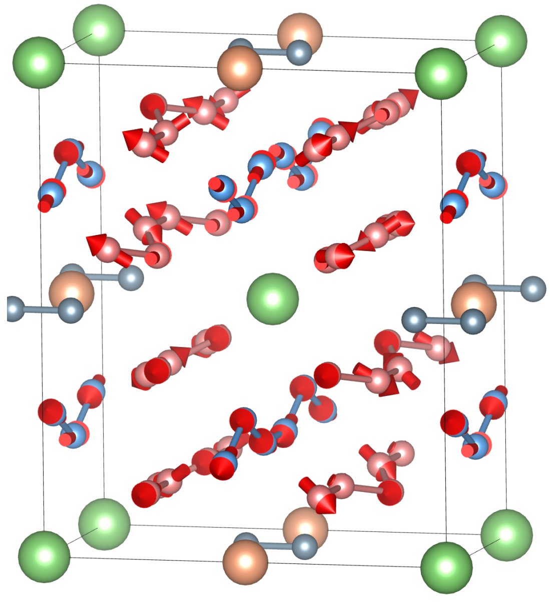

FIG. S5. Moderesolved vibrational spectra around 1500 cm^{-1} at q = (0, 0, 0).

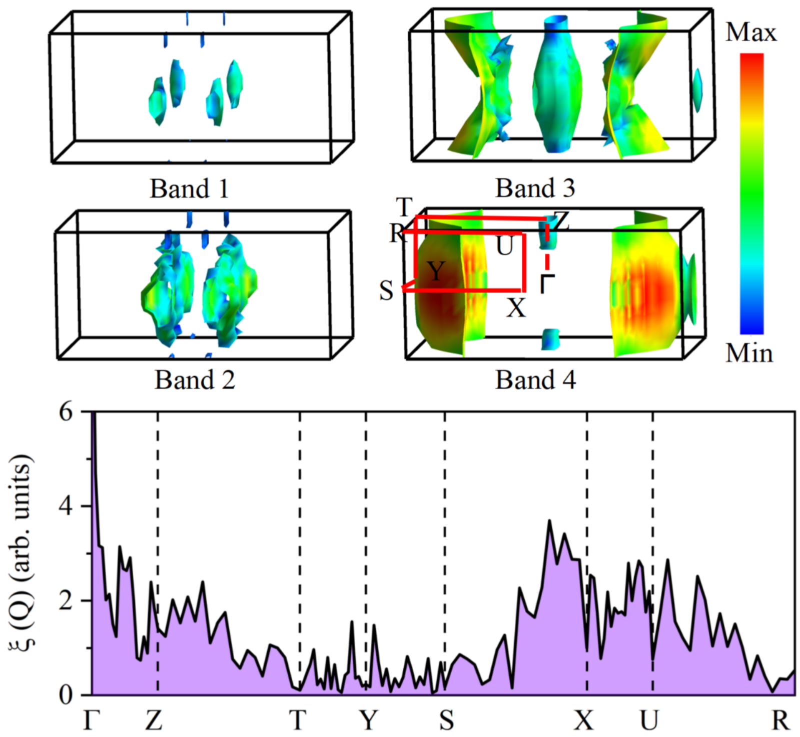

FIG. S6. The upper part of the graph shows the fermi surfaces corresponding to the four bands crossing the Fermi level for $LiHfH_{20}$ at 260 GPa. The bottom of the graph exhibits the Nesting function.

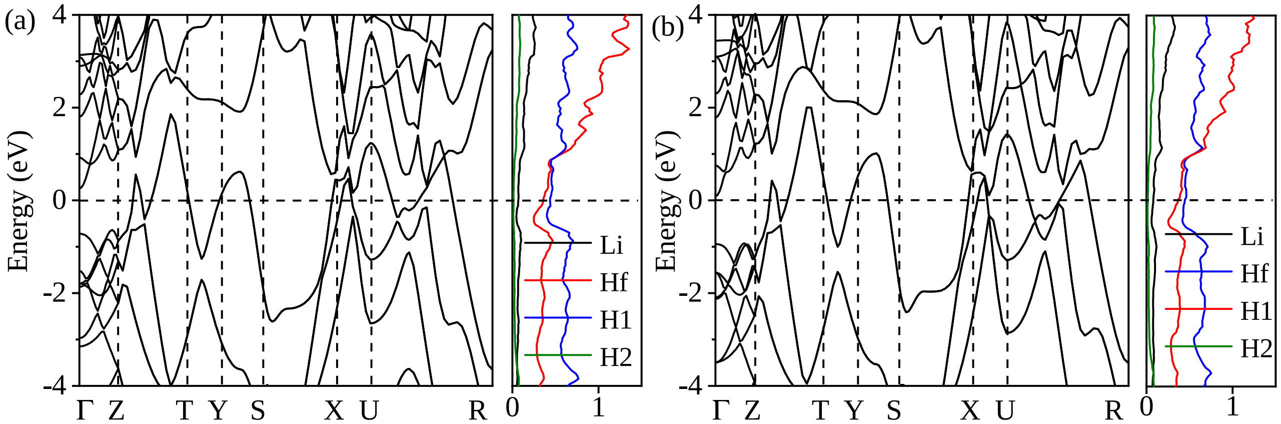

FIG. S7. Calculated electronic band structure and density of states (states/eV/cell) for $LiHfH_{20}$ at (a) 320 GPa and (b) 400 GPa, respectively.

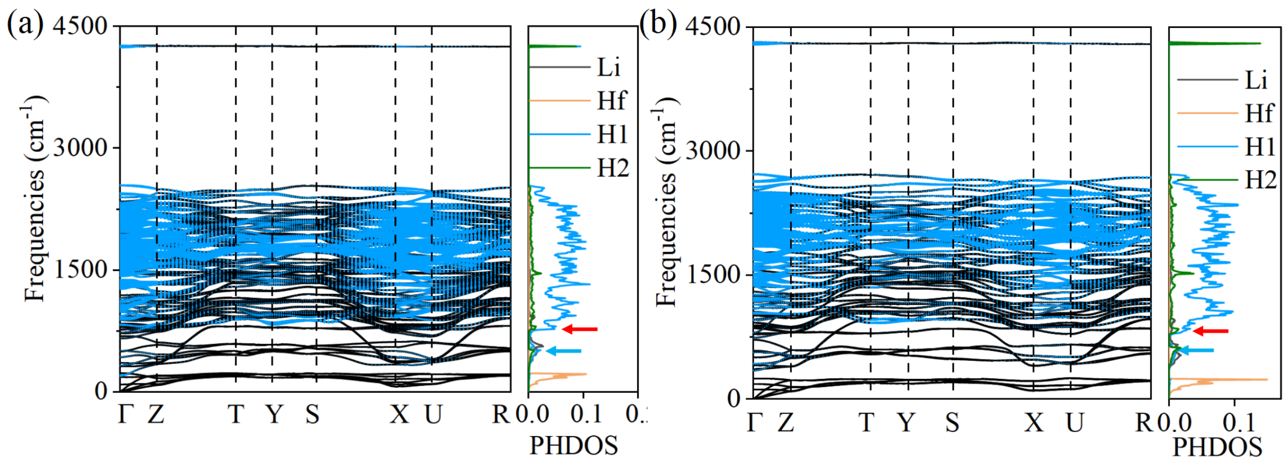

FIG. S8. Phonon dispersion, PHDOS for $LiHfH_{20}$ at (a) 320 GPa and (b) 400 GPa, respectively.

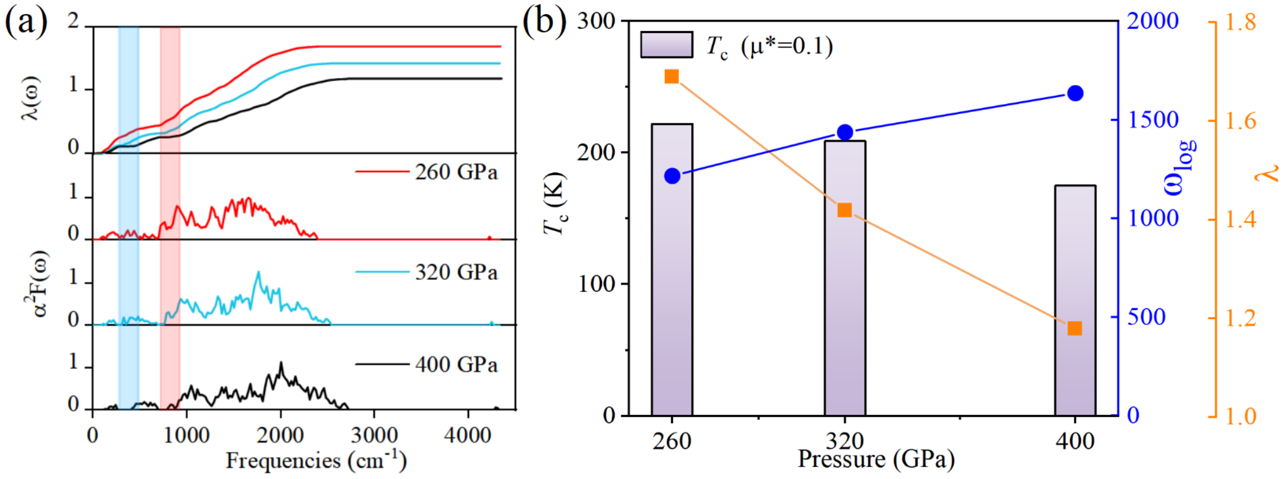

FIG. S9. Eliashberg spectral function, and the electron-phonon strength integral for $LiHfH_{20}$ at different pressures. Shaded regions in (a) show the significant contribution to λ.

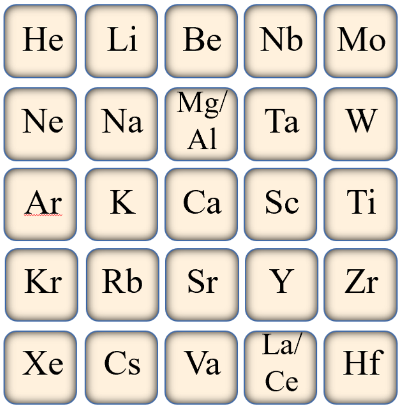

FIG. S10. The elements are what we use in the structure substitution of high-throughput screening.

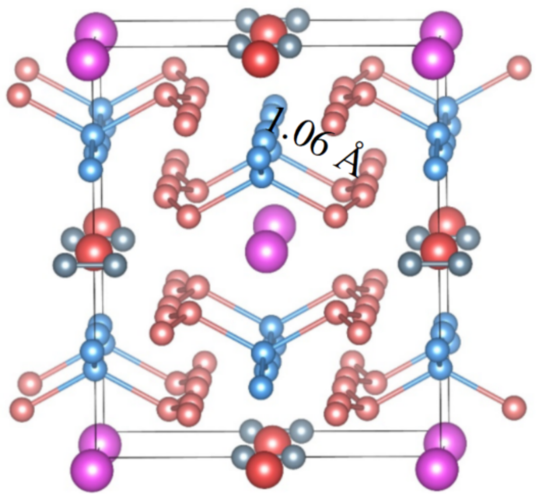

FIG. S11. Crystal structure of $BeCaH_{20}$ at 260 GPa.

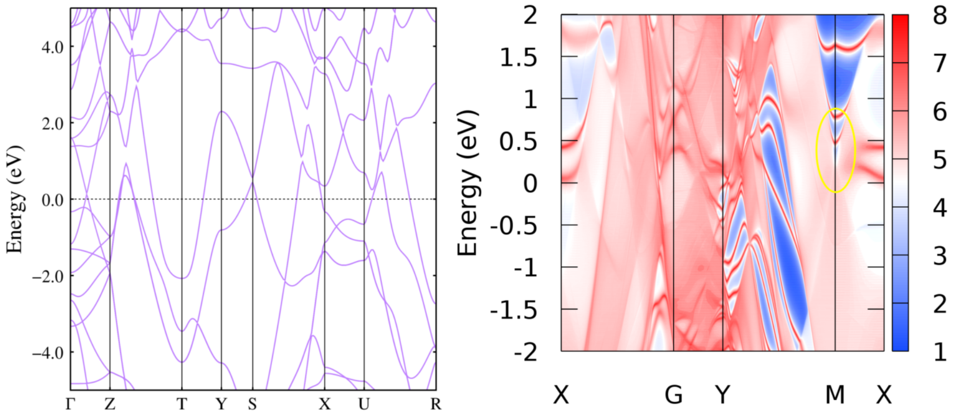

FIG. S12. Calculated band structure with SOC for $BeCaH_{20}$ at 260 GPa.

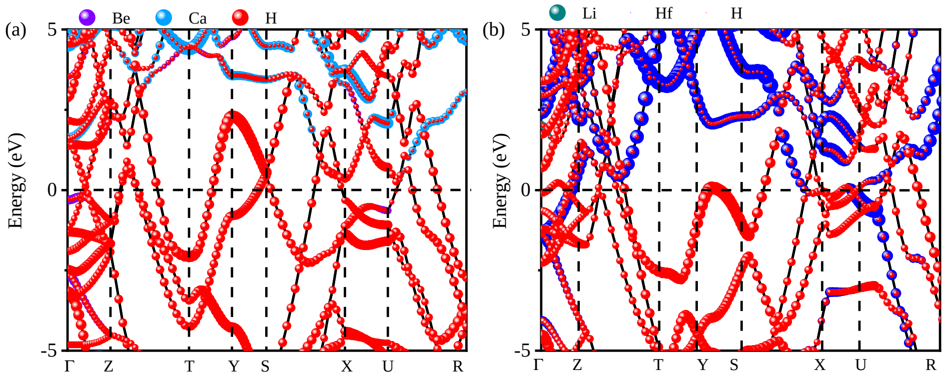

FIG. S13. Atomo-projected band structure for (a) $BeCaH_{20}$ and (b) $LiHfH_{20}$ at 260 GPa.

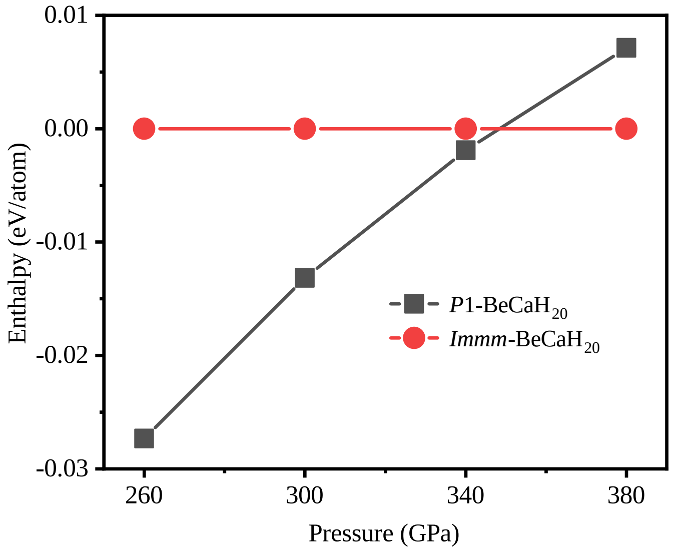

FIG. S14. Enthalpy of $P1$ and $Immm$ phase for $BeCaH_{20}$.

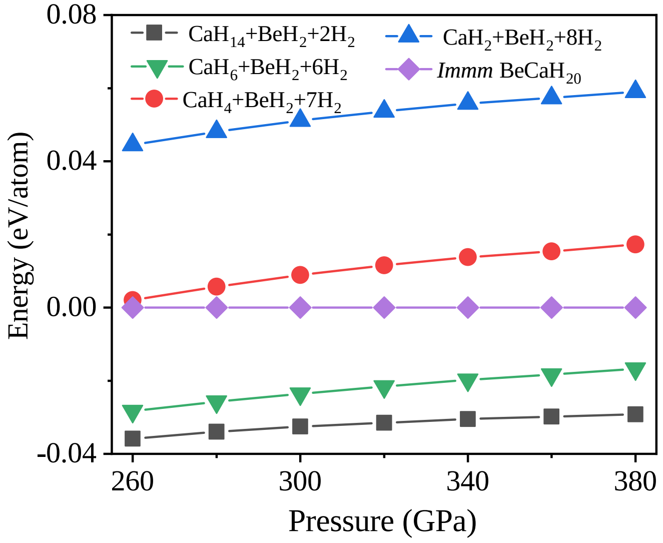

FIG. S15. Formation enthalpies of $BeCaH_{20}$ under pressuress.

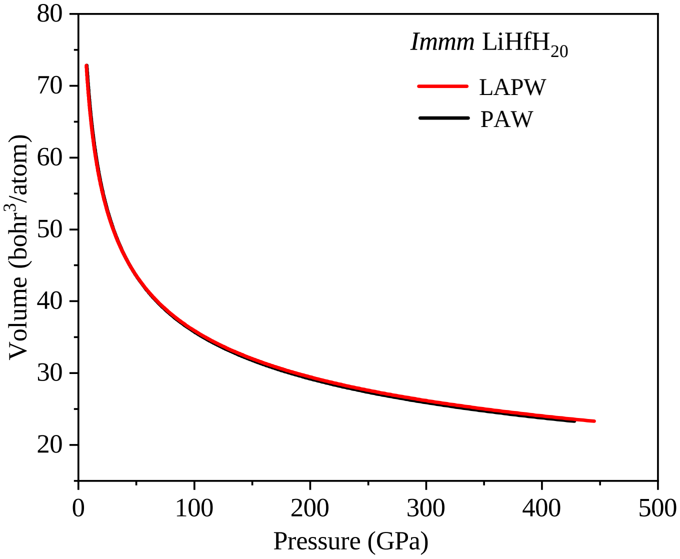

FIG. S16. Comparison of the fitted Birch-Murnaghan equation of states for $Immm$ $LiHfH_{20}$ using the PAW pseudopotentials and full-potential LAPW methods.

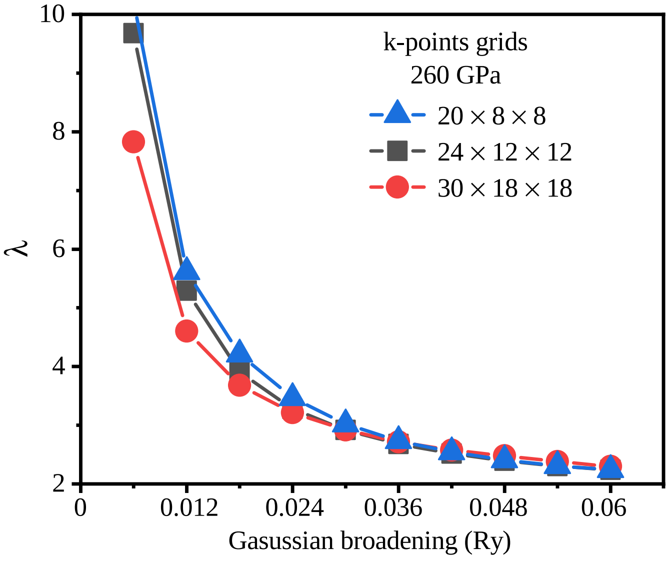

FIG. S17. Electron-phonon coupling constant λ(q) at q = (0, 0, 0) as a function of the Gaussian broadening for $BeCaH_{20}$ for different k-point grids. The 24 × 12 × 12 k-meshes os considered to be converged to the required accuracy.
